# Social restrictions due to COVID-19 and the incidence of intoxicated patients in pediatric emergency department

**DOI:** 10.1007/s11845-021-02686-0

**Published:** 2021-06-18

**Authors:** Ilari Kuitunen

**Affiliations:** 1 Institute of Clinical Medicine, Department of Pediatrics, University of Eastern Finland, Yliopistonranta 1, PL 1627, 70211 Kuopio, Finland; 2 Department of Pediatrics, Mikkeli Central Hospital, Porrassalmenkatu 35-37, 50100 Mikkeli, Finland

**Keywords:** Alcohol, COVID-19, Epidemiology, Intoxication, Lockdown

## Abstract

**Background:**

Social restrictions set due to COVID-19 decreased pediatric emergency department (ED). The aim is to report epidemiology of intoxicated patients in pediatric ED during the first and second waves of COVID-19.

**Methods:**

Data for this retrospective hospital discharge register study was gathered from January 2017 to December 2020. Patients aged <18 and intoxicated were included. Incidences are reported per 10,000 children and compared by incidence rate rations (IRRs) with 95% confidence intervals (CIs).

**Results:**

Incidence of ED visit for intoxicated patient was 65 per 10,000 in 2020 and 54 per 10,000 in 2017–2019 (IRR 1.20 CI 0.87–1.68). Incidence was lower during the lockdown compared to reference years (IRR 0.50 CI 0.17–1.44). Peak monthly incidence (12 per 10000) was recorded after lockdown in July 2020 (IRR 2.45 CI 1.01-5.92).

**Discussion:**

Based on these results, the lockdown and social restrictions did not decrease heavy alcohol or drug consumption among adolescents in Finland.

## Introduction

Finland declared COVID-19 lockdown in March like majority of European countries. Pediatric emergency department (ED) visit rates decreased immediately after the lockdown began. Schools were closed, and less visits occurred due infectious diseases [1]. Mainly the decrease was seen in the rate of pediatric primary care patients, and the rate of secondary care patients remained stable [2]. Globally, trauma-related pediatric ED visits decreased during the first wave of COVID-19 [3]. No previous studies have discussed the possible effect of social restrictions on the rates of pediatric intoxication patients. The incidence of pediatric poisonings has previously been reported to be 8 per 10,000 person-years in Finland, and alcohol was the most common cause of poisoning [4]. Alcohol is a major risk factor for injuries among adolescents in Finland [5]. The overall alcohol consumption decreased by 10% during the lockdown according to Finnish Health officials.

As the incidence of COVID-19 started to decrease in May, the restrictions were lifted in the beginning of June. The rate of pediatric patients turned to slow increase during the summer and normalized as the schools continued in August [2]. The second wave of COVID-19 started in September in Finland. During the second wave, regional restrictions were used instead of nationwide lockdown, and schools remained open. During the lockdown, a major concern in Finland was how outcast children will get along during the lockdown and school closures.

The aim of this study was to report the incidences and discharge diagnoses of intoxicated pediatric patients in pediatric ED during the first and second waves of COVID-19 in Finland and compare these to corresponding periods in three previous years.

## Methods

Data for this retrospective register-based study was gathered from Mikkeli Central Hospital, a secondary level hospital with a pediatric emergency room, which provides round-the-clock primary and secondary level care for a pediatric population of up to 19,000. The study period from 1 January 2020 to 31 December 2020 was compared with the corresponding dates in 2017–2019. Three previous years were selected as reference since the pediatric ED was established in 2017, and Mikkeli has served as the only secondary level pediatric unit in Southern Savonia region from 2016. In Mikkeli Central Hospital, the ED nurses are mandated to classify each patient as intoxicated or non-intoxicated as part of the routine primary assessment. The intoxication was determined and classified either by clinical condition or by interviewing the patient, and if alcohol intoxication was suspected, it was confirmed by breathalyzer. It must be noted that intoxication does not necessarily mean poisoning in this context, as the patients are classified as intoxicated if they were under the influence of alcohol or drugs but not suffering from poisoning. Poisonings for non-intoxicant substances were not included. To confirm the primary intoxication assessment quality of ED nurses, additional search based on the discharge diagnoses (ICD-10 diagnostic codes for intoxications: Alcohol, Drugs, Medicines) were searched, and no additional cases were found.

For our study, all patients aged under 18 years of age that were classified as intoxicated were included, and the following information was collected: date of visit, weekday, age, gender, diagnose of visit, and the need of hospitalization. Incidences were calculated per 10,000 children aged 10 to 17, as no younger patients had intoxication in our ED, and incidences were compared to mean incidence of reference years by incidence rate ratios (IRRs) with 95% confidence intervals (CIs). The chi-square test was used for categorized variables and the Mann-Whitney *U* test for continuous variables. The analyses were carried out with SPSS for Windows, version 27.0 (IBM Corp, New York, USA). ExReport software was used to gather the ED patient register report from patient discharge registers.

The chief physician of Mikkeli Central Hospital gave us permission, as the leader of local research committee, to access the discharge register data. Further approval was not required from ethical committee, and no informed consent was required according to Finnish research laws, as we studied retrospective, anonymized data and did not contact the patients or read the patient files.

## Results

**Fig. 1:**
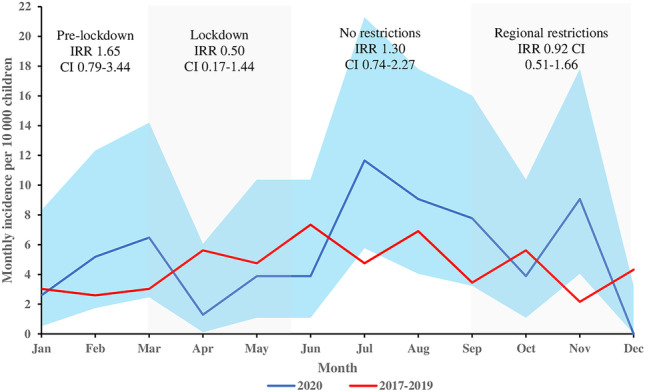
Monthly incidence per 10,000 children of emergency department visits for intoxicated patients aged under 18 years of age in Mikkeli Central Hospital. Blue line presents 2020 with 95% confidence intervals, and red line presents the monthly mean for reference years 2017–2019. Incidence rate ratios (IRRs) with 95 % CI presented for pre-lockdown, lockdown, no restrictions, and regional restriction eras

A total of 29,061 pediatric ED visits occurred in 2017–2020. Of these, 5820 (20%) were in 2020, and 23,241 (80%) were in 2017–2019. The total numbers of intoxicated patients were 50 in 2020 and 124 in 2017–2019, and higher proportion of ED visits were due intoxication in 2020 (0.8% vs 0.5%, *p*=0.01). The yearly incidence of ED visit for intoxicated patient was 65 per 10,000 children in 2020 and 54 per 10,000 in 2017–2019 (IRR 1.20 CI 0.87-1.68). Before the lockdown, the incidence of intoxicated patients was higher (IRR 1.65 CI 0.79-3.44: Fig. 1). The incidence decreased and was lower during the lockdown compared to reference years (IRR 0.50 CI 0.17-1.44: Fig. 1). After the lockdown, the incidence of intoxications increased, and the peak monthly incidence (12 per 10,000) was recorded in July 2020 (IRR 2.45 CI 1.01-5.92). Another peak in the incidence was seen in November, 9 per 10,000 (IRR 4.45 CI 1.33-13.2). Interestingly no intoxicated pediatric patients were treated in the ED in December.

The age did not differ between 2020 and the reference years, and gender distribution was similar (Table 1). The most common discharge diagnoses were alcohol intoxication followed by drug intoxication. The proportion of alcohol intoxications remained unchanged in 2020 compared to reference years. Fewer patients needed inpatient admission in 2020. Intoxication patients visited ED typically in weekends (Table 1).

**Table 1:** Background characteristics and emergency department discharge diagnoses of intoxicated pediatric patients, 2020 compared to reference years

	2020		2017–2019		*p*
	*n*	%	*n*	%	
Total visits	50	100	124	100	
Gender male^a^	25	50	67	54	0.63
Age^b^	16	2	16	2	0.78
Weekend^a^	32	64	76	62	0.74
Inpatient admission^a^	13	25	44	36	0.23
ED discharge diagnoses^a^					
- alcohol intoxication	27	54	69	56	0.19
- injury	8	16	31	25	
- drug intoxication	11	22	13	10	
- psychiatric	4	8	11	9	

^a^Cross tabulated and tested with chi-squared test

^b^Median and interquartile range, tested with Mann-Whitney *U*

## Discussion

The overall incidence of intoxicated pediatric patients was higher than in three previous years, although the lockdown and social restrictions reduced the incidence of pediatric intoxicated patients in spring 2020. A clear increase in the incidence was seen after the restrictions were lifted in summer. The school closured did not change the fact that typically, intoxications occur in weekends. Although overall ED visits due to injuries decreased in 2020, alcohol-related injuries remained near to the rates of reference years. Alcohol is a major risk factor for injuries in Finland [5]. In our study, the incidence of alcohol intoxications was higher than previously reported in Finland. This finding is explained by different search strategies, as in our hospital, the intoxication status is recorded for every patient, and in the previous studies, only diagnoses of visits were screened [4]. When interpreting these results, it should be noted that poisonings for non-intoxicating substances were not included. The main limitation for our study is the small population base which increases the uncertainty and may cause high variation in the results. The main strength is the excellent way of classifying each patient as intoxicated or non, although these classifications were assessed retrospectively. Another limitation is that the testing results for blood and urine samples were not assessed as these are not recorded to the patient discharge register. Based on these results, the lockdown and social restrictions did not decrease heavy alcohol or drug consumption among adolescents in our area. Future larger studies are needed to evaluate the long-term effects of the lockdowns and restrictions on the mental health and behavioral habits of adolescents.

## Abbreviations

CI: Confidence interval
ED: Emergency department
IRR: Incidence rate ratio
